# Sensory Input Modulates Microsaccades during Heading Perception

**DOI:** 10.3390/ijerph18062865

**Published:** 2021-03-11

**Authors:** Milena Raffi, Aurelio Trofè, Monica Perazzolo, Andrea Meoni, Alessandro Piras

**Affiliations:** 1 Department of Biomedical and Neuromotor Sciences, University of Bologna, 40126 Bologna, Italy; monica.perazzolo2@unibo.it (M.P.); andrea.meoni@unibo.it (A.M.); alessandro.piras3@unibo.it (A.P.); 2 Department of Quality of Life, University of Bologna, 47921 Rimini, Italy; aurelio.trofe2@unibo.it

**Keywords:** optic flow, self-motion perception, visual perception, eye position, eye movements, sensorimotor control, visual system

## Abstract

Microsaccades are small eye movements produced during attempted fixation. During locomotion, the eyes scan the environment; the gaze is not always directed to the focus of expansion of the optic flow field. We sought to investigate whether the microsaccadic activity was modulated by eye position during the view of radial optic flow stimuli, and if the presence or lack of a proprioceptive input signal may influence the microsaccade characteristics during self-motion perception. We recorded the oculomotor activity when subjects were either standing or sitting in front of a screen during the view of optic flow stimuli that simulated specific heading directions with different gaze positions. We recorded five trials of each stimulus. Results showed that microsaccade duration, peak velocity, and rate were significantly modulated by optic flow stimuli and trial sequence. We found that the microsaccade rate increased in each condition from trial 1 to trial 5. Microsaccade peak velocity and duration were significantly different across trials. The analysis of the microsaccade directions showed that the different combinations of optic flow and eye position evoked non-uniform directions of microsaccades in standing condition with mean vectors in the upper-left quadrant of the visual field, uncorrelated with optic flow directions and eye positions. In sitting conditions, all stimuli evoked uniform directions of microsaccades. Present results indicate that the proprioceptive signals when the subjects stand up creates a different input that could alter the eye-movement characteristics during heading perceptions.

## 1. Introduction

The optic flow fields projected on the retina allow the observer to create a neural representation of the extrapersonal space and thus to move into the environment. The first studies on the role of optic flow in self-motion perception started in the 1950s with J. J. Gibson [1,2,3]. Since then, many studies have investigated, both on animal and human models, the cortical and subcortical mechanisms responsible for heading perception. In macaques, several studies have shown precise neuronal selectivity to optic flow stimuli [4,5,6,7,8,9], to the interaction between optic flow and ocular position [10,11,12,13], and to the interaction between optic flow and other sensory signals [14,15,16]. In humans, several studies have shown that specific optic flow stimuli are important for guiding locomotion [17,18,19,20] and for the postural control [21,22,23,24,25]. The picture arising from those studies is that the analysis of the optic flow stimuli is a predominantly cortical process preparatory for specific motor actions.

Microsaccades are small eye movements produced during attempted visual fixation. Microsaccades are thus small saccades with an amplitude of less than 1° occurring 1–2 times per second [26]. The contemporary research field on microsaccades has significantly changed due to methodological advances in eye position recordings, progress in the computational modelling of eye movements, and the development of high-resolution and high-speed video-tracking systems. It is now well acknowledged that one of the primary role of microsaccades is to avoid fading of visual stimuli because of neural adaptation [27,28]. Besides that, many studies have shown that microsaccades are related to precise perceptual processes and demonstrated the interactions between the dynamics of microsaccades and cognitive processes [29,30,31,32,33,34,35]. Ziad Hafed showed that microsaccades are linked with extraretinal mechanisms that significantly alter spatial perception before the eye movement onset [36]. Considering this observation, the link between microsaccades and visual perception changes significantly; it appears to be a property of the gain modulation of visual activity by corollary discharge [36].

The great majority of the studies on microsaccades and visual perception have been performed using attentional, cued or discrimination tasks using classical visual stimuli (i.e., small bars either steady or moving across the screen), leading to a large agreement that microsaccades rates are modulated by both endogenous and exogenous attentional shifts [28]. A previous study firstly used optic flow stimuli to uncover the relationship between microsaccades and heading perception in a discrimination task, showing that microsaccade characteristics and directions are related to the correct perception of heading [37]. In the present work, we moved forward, analyzing the microsaccade characteristics during the view of radial optic flow stimuli, given that such stimuli attract attention toward the focus of expansion [38]. In this experiment we combined optic flow stimuli and eye positions to simulate different headings. The rationale of the protocol arises from the fact that the visual perception of self-motion is mainly due to the optic flow fields. However, in daily life, during locomotion, the eyes continuously scan the environment; thus, the gaze is not always directed to the focus of expansion of the optic flow field. Such eye movements change the retinal position of the focus of expansion with respect to the fovea, likely increasing microsaccade generation, given that the visual system uses microsaccades to heighten information acquisition from informative regions of the visual field [39]. We sought to investigate whether the microsaccadic activity was modulated by eye position during the view of radial optic flow stimuli and if the view of different optic flow stimuli changes the microsaccade characteristics and directions. Furthermore, we were interested in studying if a different input signal may influence the oculomotor activity during self-motion perception. This interest arises from the consensus in the activity of the superior colliculus, which is known to be involved in integrating multisensory signals to serve crucial functions in guiding the motor responses toward visual stimuli in space [40]. The superior colliculus is involved in visually guided behaviors in order to build up unified, coherent, and meaningful sensory perceptions during self-motion in space [41]. The superior colliculus processes signals conveying head-re-body position, suggesting that collicular neurons contribute to a displacement to position transformation for oculomotor control [42]. Neurons in the superior colliculus integrate visual, auditory and somatosensory inputs from subcortical and cortical sensory structures [43]. From these premises, we decided to perform the same experiment in two experimental conditions: when subjects viewed the optic flow stimuli while standing in front of a screen, and when subjects viewed the optic flow stimuli sitting in front of a screen. We hypothesized that the presence of the proprioceptive input when the subjects stand up creates a different input that could alter the eye-movements characteristics during heading perception, given that while standing there is an increased cognitive load with respect to sitting.

## 2. Materials and Methods

The experiments were performed on 19 healthy volunteers, 4 females and 15 males, who participated in two different experimental sessions carried out on two different days. In the first session, we recorded eye movements when the subjects were standing, while in the second session the subjects were seated. Three participants dropped out between sessions, so data were recorded on 19 people in standing conditions and 16 people in sitting conditions. Direct comparisons have been performed on 16 subjects. The subjects’ age ranged from 19 to 38 years (average 25.6 ± 4.9 SD). All participants had normal vision. Before the beginning of the experiment, the hand and foot laterality of each participant was assessed by a laterality questionnaire [44,45] using the following formula:

[(right preference−left preference)/(right preference + left preference)] × 100(1)

A positive index indicates a right dominance, while a negative index indicates a left dominance. The rationale to compute a laterality index was to correlate the microsaccade directions with the dominant side to elucidate the potential mechanisms for motor control.

Each participant read and signed a written informed consent before participating in the study. The study protocol was approved by the Institutional Bioethic Committee of the University of Bologna. The experiments were performed in accordance with the ethical standards laid down in the 1964 Declaration of Helsinki.

### 2.1. Optic Flow Stimuli

In this experiment, we presented the same stimuli used in a previous study [24]. Briefly, the stimuli were made by 1155 white dots (1.3 cd/m^2^, size 0.4°) presented full-field on a translucent screen that covered 135 × 107° of the visual field. Dots moved a perceived speed of 5°/s. All recordings were performed in a dark room with dark walls. Each participant was instructed to fixate on a fixation point (FP) of 0.6° size. The binocular eye position of each subject was recorded both in standing and sitting condition. The screen height was adjusted for each subject and each condition, to ensure that the FP was in the primary position. To study the influence of the microsaccades during optic flow stimulation, we modified the speed of the dot pattern and the gaze direction, changing the FP. Specifically, the FP was presented in one of three positions along the horizontal axis (in the center, 15° to the left, or 15° to the right). The FP was always concentric with the focus of expansion of the optic flow stimulus. The dot speed was accelerated to the left or to the right hemifield to simulate different headings at different angles of gaze [46].

**Figure 1:**
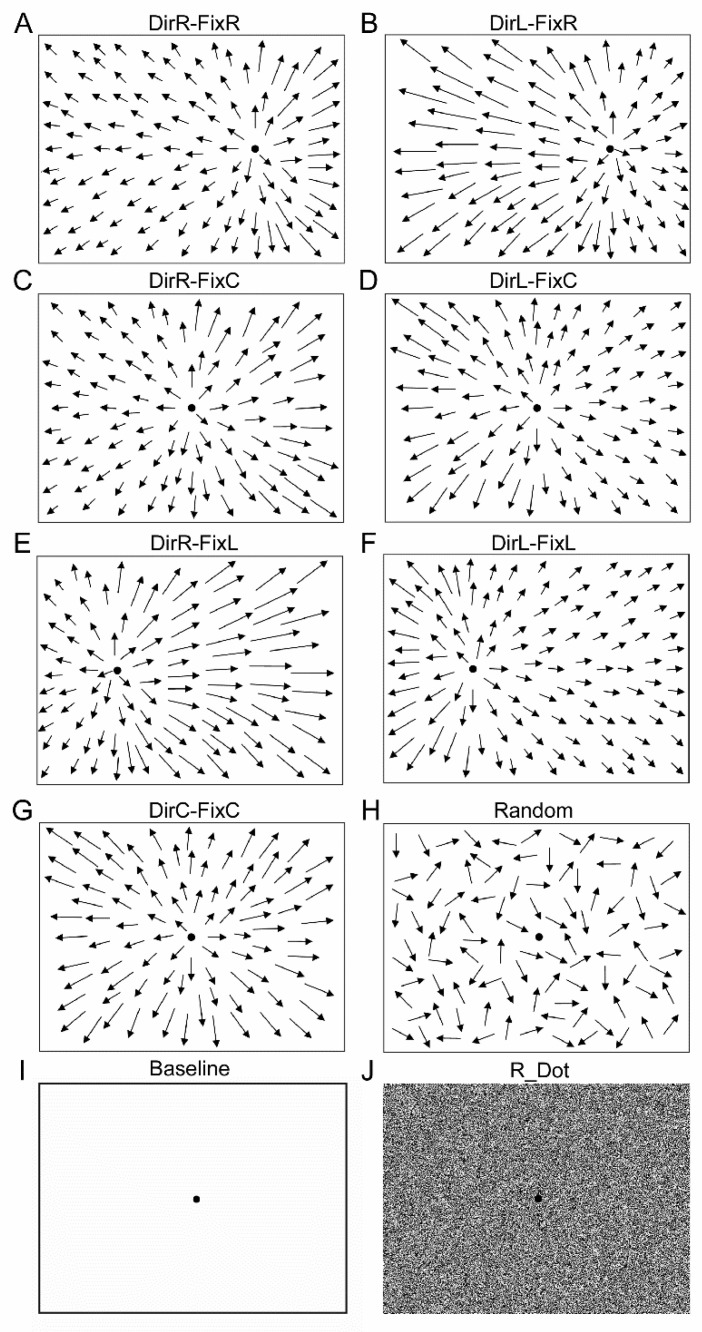
Optic flow and control stimuli. (**A**) Fixation point (FP) to the right and dots accelerated to the right simulated heading direction to the right while fixation was to the right (DirR-FixR). (**B**) FP to the right and dots accelerated to the left simulated heading direction to the left while fixation was to the right (DirL-FixR). (**C**) FP to the center and dots accelerated to the right simulated heading direction to the right while fixation was straight ahead (DirR-FixC). (**D**) FP to the center and dots accelerated to the left simulated heading direction to the left while fixation was straight ahead (DirL-FixC). (**E**) FP to the left and dots accelerated to the right simulated heading direction to the right while fixation was to the left (DirR-FixL). (**F**) FP to the left and dots accelerated to the left simulated both heading and fixation to the left (DirL-FixL). (**G**) Radial expansion concentric with the FP simulated heading direction and fixation straight ahead (DirC-FixC). (**H**) Random dot motion (Random). (**I**) Baseline condition (Baseline). (**J**) Static random dots (R_Dot). Arrows represent the velocity vectors of moving dots.

Figure 1 shows the stimuli used: DirR-FixR had FP to the right and dots accelerated to the right simulated heading direction to the right while fixation was to the right (Figure 1A). DirL-FixR had FP to the right and dots accelerated to the left to simulate heading direction to the left while fixation was to the right (Figure 1B). DirR-FixC had FP to the center and dots accelerated to the right to simulate heading direction to the right while fixation was straight ahead (Figure 1C). DirL-FixC had FP to the center and dots accelerated to the left to simulate heading direction to the left while fixation was straight ahead (Figure 1D). DirR-FixL had FP to the left and dots accelerated to the right to simulate heading direction to the right while fixation was to the left (Figure 1E). DirL-FixL had FP to the left and dots accelerated to the left to simulate both heading and fixation to the left (Figure 1F). DirC-FixC had dots expanding radially concentric with the FP to simulate heading direction and fixation straight ahead (Figure 1G). Three stimuli were used as controls. Random consisted of random dot motion (Figure 1H); Baseline consisted of simple fixation on a dark screen (Figure 1I); and R_Dot consisted of static random dots (Figure 1J). Optic flow stimuli were made using the Matlab psychophysical toolbox (The Mathworks Inc. Natick, MA, USA). We recorded 5 trials, i.e., 5 repetitions, for each stimulus; thus, each subject performed 50 trials in standing condition and 50 trials in sitting condition.

### 2.2. Eye Movements and Eye Position Recordings

We recorded the horizontal and vertical eye movements using the EyeLink video-based eye tracking system (EyeLink^®^ II, SR Research Ltd., Mississauga, Canada). This system consists of two miniature cameras mounted on a leather-padded headband. Pupil tracking was performed at 500 samples/s, with high spatial resolution (<0.005°) and low noise (<0.01°). At the beginning of each recording session, we performed the eye tracking calibration in which the experimental subjects were instructed to fixate a target presented in random order in a nine-point 25 × 25° square grid. After a correct camera calibration, the data were validated and drift correction was executed by applying a corrective offset to the raw eye-position.

### 2.3. Data Analysis

Microsaccades are small eye movements, which occur during prolonged visual fixation. By definition, microsaccade amplitude is less than 1° and the main sequence curve (amplitude vs peak velocity) follows the same trend of large saccades [47]. To identify microsaccades, we developed an algorithm based on that of Otero-Millan [48]. To reduce the amount of potential noise, we considered only binocular microsaccades during at least 3 data samples (6 ms). Trials with incorrect fixations, eye blinks, or behavioural errors were discarded. We removed portions of data when very fast decreases and increases in the pupil area occurred (>50 units/sample). Such periods are likely semi-blinks where the pupil is never fully occluded. We also ignored the 200 ms before and after each blink/semi-blink to eliminate the initial and final parts where the pupil was still partially occluded [49].

Microsaccade amplitude, duration, direction, rate, and peak velocity were first calculated for each subject, in each trial and in each condition separately. Then, values for all subjects in each condition and trial were averaged. Microsaccade rates were calculated considering only the time spent in fixation periods: the total number of microsaccades for each subject in each trial was divided by the total time spent in fixation during that trial.

A repeated measure ANOVA was performed separately, to analyze microsaccade rates, amplitudes, durations, and peak velocities, with stimuli (DirR-FixR, DirL-FixR, DirR-FixC, DirL-FixC, DirR-FixL, DirL-FixL, DirC-FixC, Random, Baseline, R_Dot), trials (from 1 to 5) and condition (standing or sitting) as the within-subject factors. Analysis of variance was performed with SPSS^®^ 22.0 statistical package (SPSS version 22.0 software IBM, Chicago, IL, USA). Results were considered significant at p < 0.05. Multiple comparisons have been analyzed in each parameter.

Microsaccade directions were computed using the algorithm developed by Otero-Millan [48]. Such algorithm produces a representation of the interval 0–90° in the fourth quadrant, so we applied an angular rotation to each vector to bring the representation 0–90° into the conventional polar coordinate reference system. To study a possible interaction between microsaccades and optic flow directions, we used circular statistics (Oriana^®^ 4.0 for Windows, Kovach Computing Services, Anglesey, Wales).

Circular statistics are statistical techniques for use with data on an angular scale. Such techniques deal with angular directions or rotations. These statistical methods are required for the analysis of angular data; for example, 0° and 360° are identical angles, but 180° is not the average of 2° and 358°. In the present data, the uniformity of the mean vectors distribution was assessed with the Rayleigh test of uniformity and results were considered significant at *p* < 0.05.

## 3. Results

The analysis of the laterality test showed that 16 subjects were right-handed and 3 subjects were left handed. Answers to the laterality questionnaire for the right-handed subjects resulted in values ranging from 68.42 to 100, indicating a strong right laterality. Values for the left-handed subjects were −36,84, −89,47, and −100, indicating a strong left laterality for two subjects.

**Figure 2:**
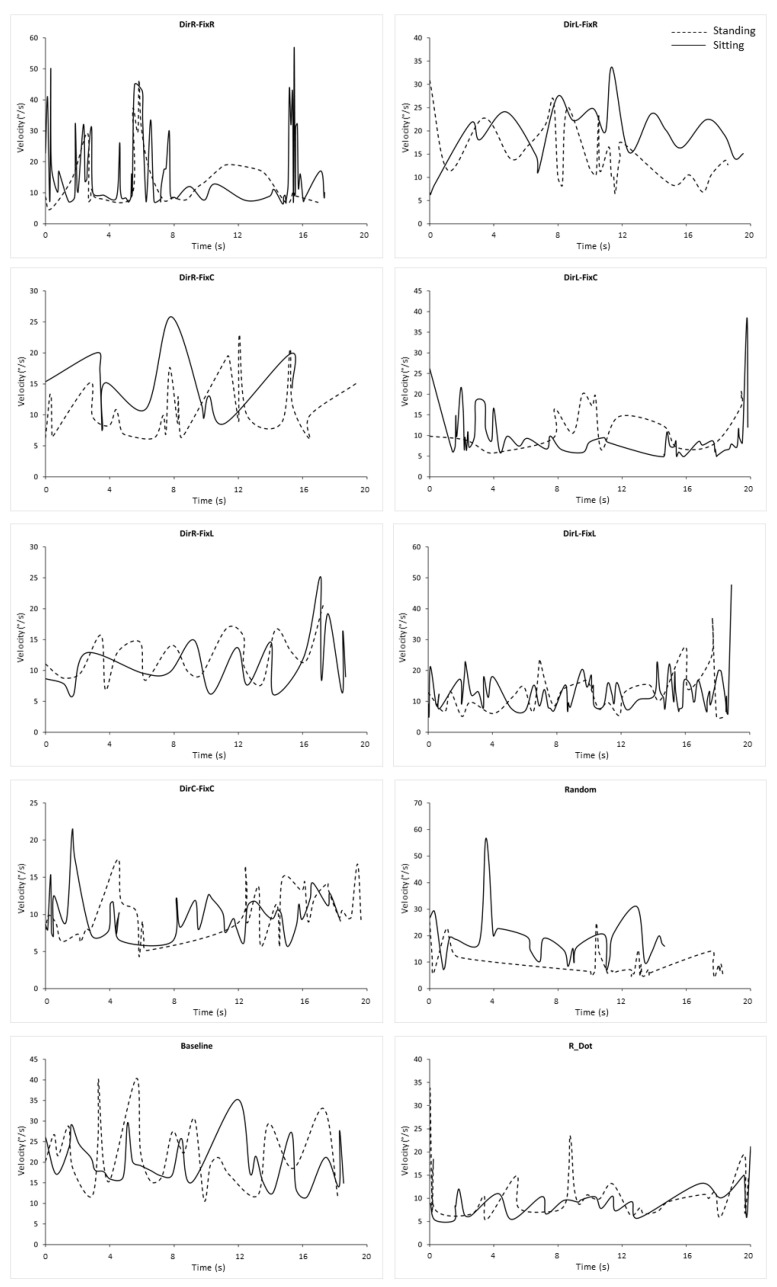
Waveforms of microsaccade velocity in all stimuli in both conditions. Each line in each diagram represents the waveform of all microsaccades recorded in an exemplary subject and trial. DirR-FixR: subject 10, trial 1. DirL-FixR: subject 16, trial 5. DirR-FixC: subject 05, trial 3. DirL-FixC: subject 19, trial 2. Baseline: subject 03, trial 1. DirR-FixL: subject 18, trial 1. DirL-FixL: subject 10, trial 3. DirC-FixC: subject 05, trial 3. Random: subject 06, trial 4. Baseline: subject 03, trial 1. R_Dot: subject 19, trial 5. Conventions are as in Figure 1.

**Figure 3:**
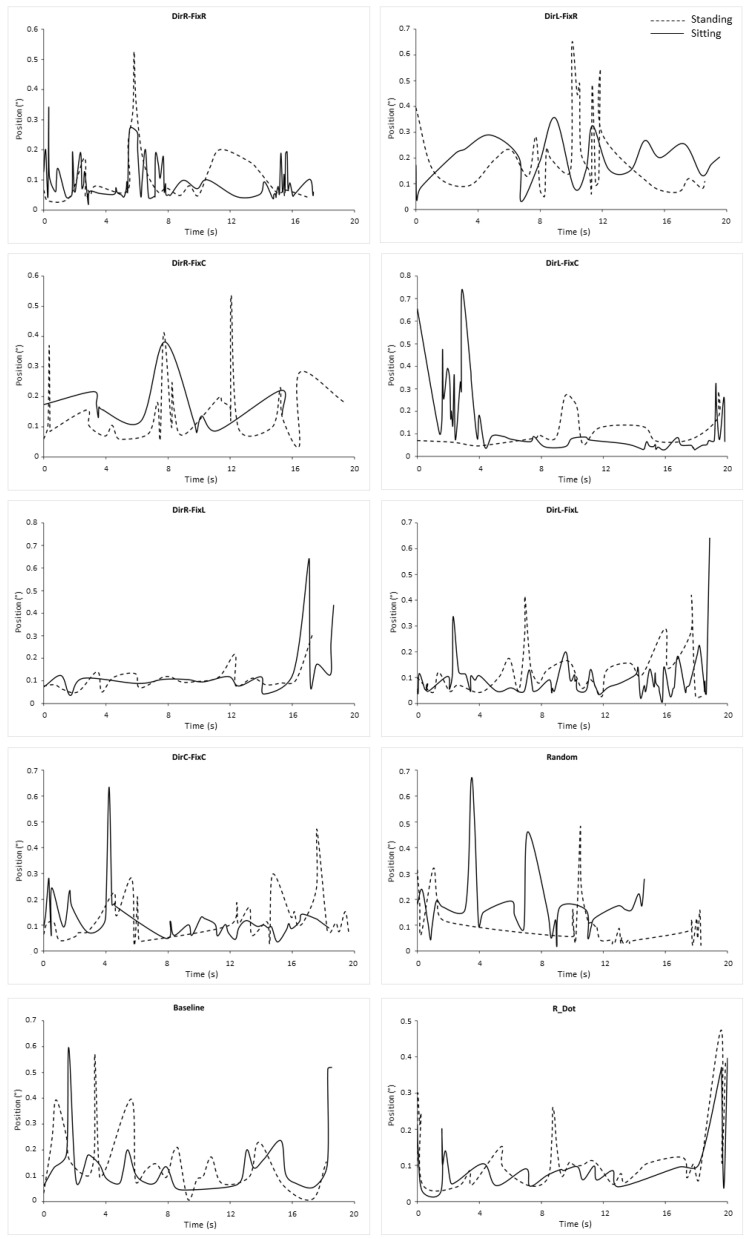
Waveforms of microsaccade positions in all stimuli in both conditions. Each line in each diagram represents the waveform of all microsaccades recorded in an exemplary subject and trial. DirR-FixR: subject 10, trial 1. DirL-FixR: subject 16, trial 5. DirR-FixC: subject 05, trial 3. DirL-FixC: subject 19, trial 2. Baseline: subject 03, trial 1. DirR-FixL: subject 18, trial 1. DirL-FixL: subject 10, trial 3. DirC-FixC: subject 05, trial 3. Random: subject 06, trial 4. Baseline: subject 03, trial 1. R_Dot: subject 19, trial 5. Conventions are as in Figure 1.

To verify the identity of the eye movements, to avoid including potential nystagmus, we plotted velocity (Figure 2) and position (Figure 3) waveforms of exemplary microsaccades for both conditions in each stimulus. To allow comparisons, plots of Figure 2 and Figure 3 were taken from the same trial and subject.

### 3.1. Main Sequence

**Figure 4:**
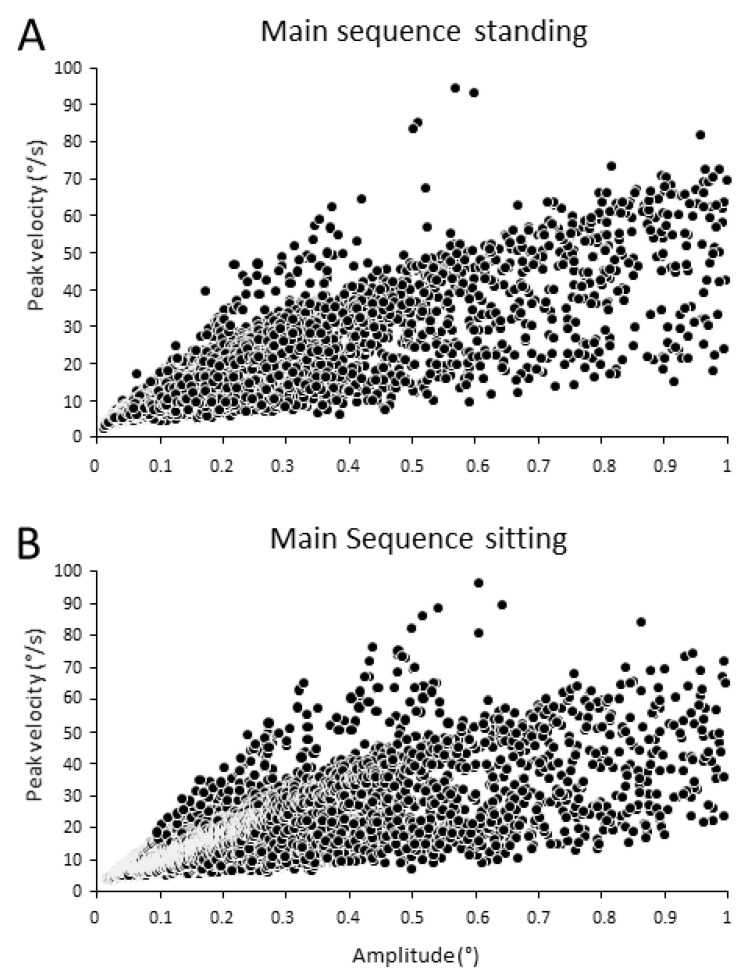
Microsaccade main sequence. (**A**) Main sequence when subjects were standing. Total number of microsaccades: 12,078. (**B**) Main sequence when subjects were seated. Total number of microsaccades: 13,938.

The total number of analyzed trials for standing condition was 950, while for sitting it was 800. Figure 4 shows the main sequence, i.e., the amplitude–peak velocity relationship of all microsaccades during standing (Figure 4A) and sitting (Figure 4B), in all stimuli. This relationship followed the trend of large saccades. In standing position, the total number of microsaccades was 12,078, while in sitting position the total number of microsaccades was 13,938.

### 3.2. Microsaccades Duration

**Figure 5:**
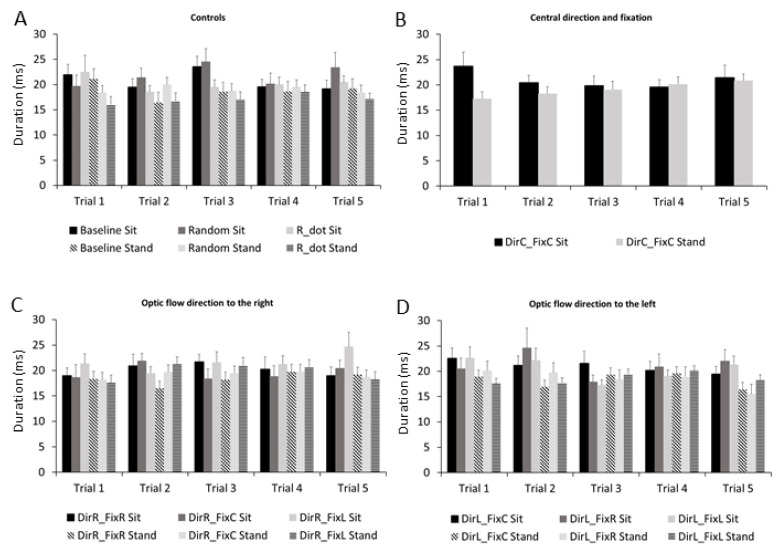
Frequency histograms of microsaccade durations in all stimuli across trials in both sitting and standing conditions. (**A**) Controls: Baseline, R_dot, Random. (**B**) Central direction and fixation: DirC-FixC. (**C**) Optic flow direction to the right: DirR-FixR, DirR-FixC, DirR-FixL. (**D**) Optic flow direction to the left: DirL-FixR, DirL-FixC, DirL-FixL. Data are reported as mean ± SE. Conventions are as in Figure 1.

The ANOVA performed on the microsaccade duration showed the main effect of stimulus (F_1,9_ = 2.21, *p* = 0.025, η_p_^2^ = 0.12) and an interaction effect of trial × stimuli (F_1,36_ = 1.56, *p* = 0.021, η_p_^2^ = 0.09). Figure 5 shows the changes in microsaccade duration through the entire experiment for each stimulus from trial 1 to trial 5. The microsaccade duration increased in almost all stimuli across trials. Control stimuli (Figure 5A) and stimuli with optic flow directions to the right (Figure 5C) showed the highest duration.

### 3.3. Microsaccades Peak Velocity

**Figure 6:**
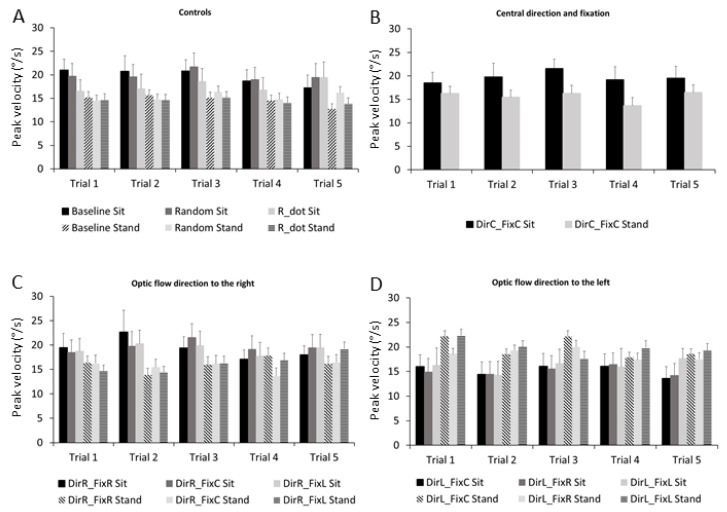
Frequency histograms of microsaccade peak velocities in all stimuli across trials in both sitting and standing conditions. (**A**) Controls: Baseline, R_dot, Random. (**B**) Central direction and fixation: DirC-FixC. (**C**) Optic flow direction to the right: DirR-FixR, DirR-FixC, DirR-FixL. (**D**) Optic flow direction to the left: DirL-FixR, DirL-FixC, DirL-FixL. Data are reported as mean ± SE. Sitting condition is reported in black, standing condition in grey. Conventions are as in Figure 1.

The ANOVA performed on the microsaccade peak velocities showed a main effect for trial (F_1,4_ = 3.29, *p* = 0.017, η_p_^2^ = 0.18), stimuli (F_1,9_ = 5.14, *p* < 0.001, η_p_^2^ = 0.25) and trial × stimuli interaction effects (F_1,36_ = 1.57, *p* = 0.02, η_p_^2^ = 0.09) (Figure 6). The Bonferroni pairwise comparison showed few stimuli differences (Table 1). 

The microsaccade peak velocity decreased in many control stimuli (Figure 6A), while it showed a fluctuant effect for the majority of the optic flow stimuli (Figure 6B–D). 

### 3.4. Microsaccades Rate

**Figure 7:**
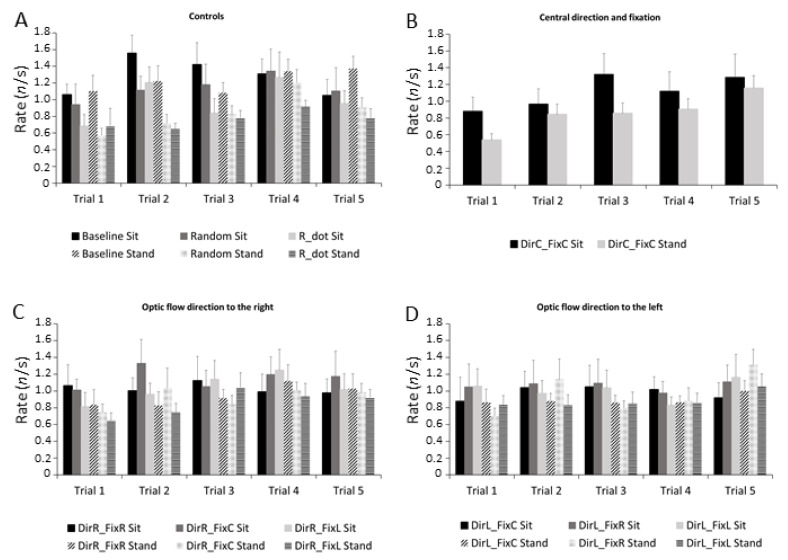
Frequency histograms of microsaccade rates in all stimuli across trials in both sitting and standing conditions. (**A**) Controls: Baseline, R_dot, Random. (**B**) Central direction and fixation: DirC-FixC. (**C**) Optic flow direction to the right: DirR-FixR, DirR-FixC, DirR-FixL. (**D**) Optic flow direction to the left: DirL-FixR, DirL-FixC, DirL-FixL. Sitting condition is reported in black, standing condition in grey. Data are reported as mean ± SE. Conventions are as in Figure 1.

The ANOVA showed a significant main effect for stimuli (F_1,9_ = 3.33, *p* = 0.007, η_p_^2^ = 0.52) and trial (F_1,4_ = 3.85, *p* = 0.031, η_p_^2^ = 0.56). The microsaccade rate increased in almost all stimuli across trials. Control stimuli (Figure 7A), central direction and fixation (Figure 7B) and stimuli with optic flow direction to the right (Figure 7C) caused the greatest rate increment, while stimuli with optic flow direction to the left (Figure 7D) elicited a moderate rate increment. 

### 3.5. Microsaccades Directions

**Figure 8:**
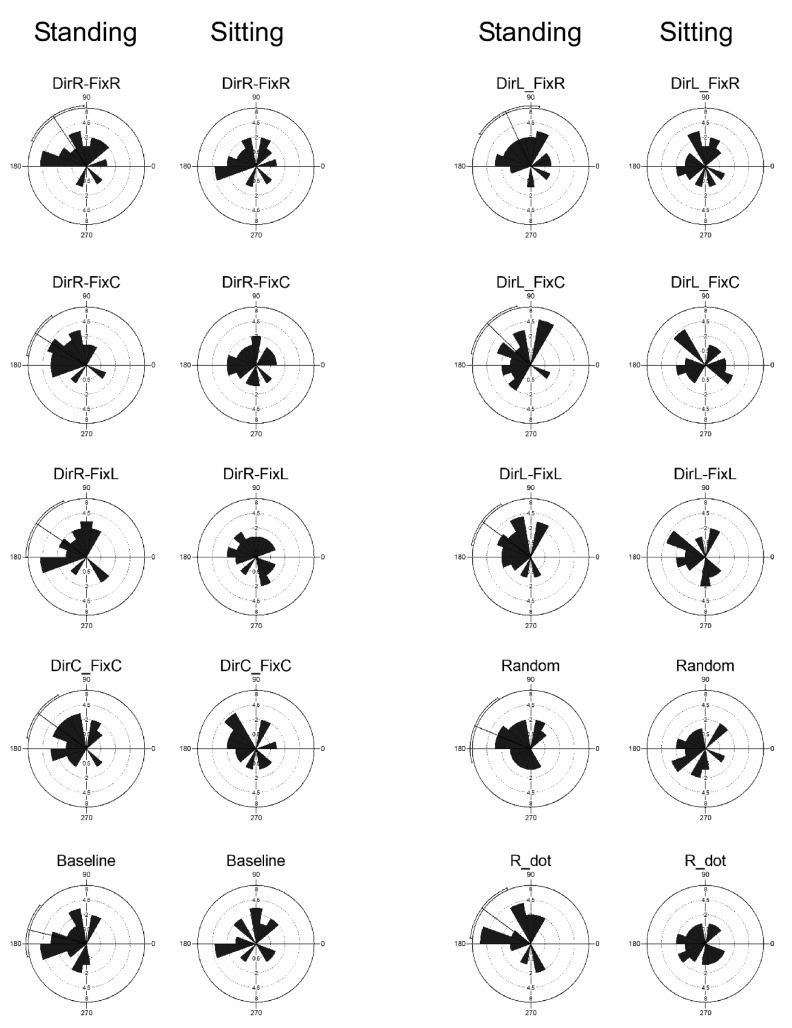
Distributions of microsaccade directions in all stimuli in both sitting and standing conditions. Mean vectors were computed for each trial. Solid line crossing each diagram indicates the significant mean vectors, curved line outside the circle indicates the circular SD. Bars are 20° width. Conventions are as in Figure 1.

Our hypothesis was that the microsaccade directions might be influenced by the combined interaction of optic flow and eye position and by the presence of a proprioceptive input. Figure 8 shows the distribution of microsaccade directions in all stimuli in both conditions. Rose diagrams are shown paired for standing and sitting. In standing condition, all stimuli evoked a non-uniform distribution of microsaccade directions: Baseline *p* < 0.001, mean vector 166°; DirC-FixC *p* < 0.001, mean vector 143°; DirR-FixC *p* < 0.001, mean vector 147°; DirR-FixR *p* = 0.003, mean vector 123°; DirR-FixL *p* = 0.007, mean vector 146°; DirL-FixC *p* = 0.005, mean vector 136°; DirL-FixR *p* = 0.005, mean vector 114°; DirL-FixL *p* < 0.001, mean vector 143°; Random *p* = 0.009, mean vector 157°; R_dot *p* = 0.003, mean vector 143° (Rayleigh test of uniformity). However, in sitting condition, all stimuli evoked a uniform distribution of microsaccade directions. It has to be noted that in standing condition, the mean significant vectors were always located in the upper-left quadrant of the visual field, which was uncorrelated with the optic flow stimulus direction and eye position. 

## 4. Discussion

The aim of the present study was to elucidate the microsaccade characteristics during the passive view of optic flow stimuli to verify the potential role of microsaccades during heading perception. To uncover the role of the sensory systems, we used radial optic flow stimuli with different angles of gaze and control stimuli in two experimental conditions: standing and sitting. By the use of ANOVA and circular statistics, microsaccade duration, peak velocity, rate, and direction showed significant effects, while microsaccade amplitude did not show any significant effect.

### 4.1. The Effect of Optic Flow Stimuli on Microsaccades

It is well known that the preferred locus of fixation is much smaller than the fovea [50], and microsaccades bring task-relevant visual targets to the preferred subregion of the fovea, improving visual acuity [51,52]. Visual perception can be altered before microsaccades, and perceptual/motor responses are suppressed after microsaccades [36,53,54,55,56]. Microsaccade generation, direction, and timing have been correlated to the appearance of sensory stimuli, attentional processes, and the degree of active fixation [29,54,56,57,58]. The present study was thus designed to elucidate whether such microsaccade characteristics change during the view of different optic flow stimuli with different structures and directions. The microsaccade rates and durations increased in almost all stimuli across trials (Figure 5 and 7); meanwhile, the microsaccades peak velocity decreased in the majority of the control stimuli (Figure 6).

Microsaccade behaviors are strongly influenced by high cognitive activities [59,60]. Gao et al. [61] showed that nonvisual cognitive processing can suppress microsaccade rates, and that the extent of such suppression is related to the task difficulty. Later, Xue at al. [62] showed that high perceptual load suppresses the rate and amplitude of microsaccades, suggesting that the microsaccades’ behavior could be an effective indicator of the perceptual load. The opposite trend of microsaccades’ behavior visible in our results is explicable with the familiarity of the task. The subjects of the present study performed five trials of the same stimulus. It is possible to hypothesize that the rate and duration of the microsaccades increased because, after the first presentation, there was nothing to explore in the stimuli. Such lack of saliency reflects a very low cognitive activity. Our results suggest that heading perception increases microsaccades’ rate and duration, and that the extent of such an increase is related to the number of stimulus presentations. To better confirm this finding, future studies should include the appearance of attentional cues within the optic flow stimuli, so as to dissociate the pure heading perception mechanisms from the involvement of attentional mechanisms during heading perception.

### 4.2. The Role of the Proprioceptive Input on Microsaccades Characteristics and Directions

In the present study, the two experimental conditions differed only for the proprioceptive and vestibular input; in the sitting condition, such input was robustly reduced. As shown in a previous study [38], the view of radial expanding optic flow patterns attracts attention toward the focus of expansion. When the attention is directed toward a specific point in the peripheral visual field, the direction of the microsaccades indicates the focus of attention. In this experiment, we chose to use a series of global stimuli that did not require the subject to shift attention to the peripheral visual field, which enabled us to dissociate the microsaccade motor response and the perception of the stimulus. In standing condition, the analysis showed that all stimuli evoked a non-uniform distribution of microsaccade directions in the upper-left quadrant of the visual field (Figure 8). The various directions of self-motion and the different gaze angles did not show any modulatory effect on the microsaccades, indicating that attention was likely always centered on the focus of expansion and was never widespread. A very different situation was observed when the subjects were sitting on a chair looking at the optic flow, where all stimuli evoked uniform microsaccade directions (Figure 8). Present results indicate that while standing, when the attention is located toward the focus of expansion with full sensory input, the cortical processing of optic flow perception may drive the oculomotor response toward the postural response; the mean vectors of microsaccade directions were significantly clustered in the upper-left visual field. The opposite situation occurred when the subjects were sitting and the sensory input diminished, because the mean vectors were uniformly distributed.

Although a strong effect, the finding that the microsaccades are always directed toward the upper-left visual field in a standing position during heading perception, is somehow surprising. Our hypothesis for such phenomenon is that in the absence of peripheral attention during self-motion perception, the oculomotor microsaccadic response matches the postural response. In a previous study, we showed that visual feedback differently influences the neural control of body sway; thus, the neural activity seems to provide the motor system with different afferent inputs in response to disturbances of body balance [25]. It seems that the neural resources to process information related to heading perception during multisensory integration do not allow the generation of exploratory microsaccades, which instead happens when the sensory input is strongly reduced (i.e, sitting condition). The present results also open an important question: why are the microsaccade directions directed toward the upper-left visual field? According to our previous results [24,25], the microsaccade directions could reflect the body sway oscillation in response to the optic flow field. In a previous study, we recorded the body sway in a group of subjects using the same stimuli used in this experiment; results showed a significant body oscillation toward the upper-left space (Figures 3 and 4 of [24]). Such oscillations may arise from the laterality, because the subjects were right-handed and right-footed, or from the motor response evoked by the optic flow stimuli, supporting the view that a person has their own way to stand using peculiar motor coordination dynamics to control posture [24].

Reed-Jones and co-workers performed an experiment in which subjects were immersed in a virtual environment, which simulated walking down a hallway and turning a corner [63]. In half of the trials, the subjects were required to fixate on a static target placed in the middle of the screen, while in the remaining trials the gaze was unconstrained. Results of their experiment showed that gaze fixation on a stationary target suppressed the anticipatory steering responses. Although postural adjustments were still observed during constrained gaze trials, such adjustments were significantly smaller than trials in which gaze was unconstrained. These findings indicate that gaze redirection is a prerequisite for the initiation of a pre-programmed motor action, suggesting that the postural responses are closely linked to the oculomotor control processes within the central nervous system. In our experiment, and for the entire trial duration, the subjects’ gaze was always directed to the fixation point.

We are aware that this experimental condition does not reflect real oculomotor behavior, because in everyday life, the eyes are usually not fixated on an object for more than a few seconds. However, as already stated by Hafed et al. [34], investigating the role of microsaccades in experiments that require fixation is necessary because these experiments themselves allow inferences to be made about vision and cognitive processes.

## 5. Conclusions

The results show that microsaccade rate, amplitude and peak velocity are strongly influenced by the combination of optic flow and eye position, while microsaccade directions are only influenced by standing or sitting conditions. During standing, the microsaccade directions were significantly clustered toward the upper-left quadrant of the visual field, while during sitting the microsaccade directions were uniformly distributed. According to Hafed et al. [34], the role of microsaccades in modulating neuronal responses in the visual system is more sophisticated than a simple retinal refresh, extending to changes in response gain, spatial representations, and possibly neural coding. The present results open new horizons on the study and role of the microsaccadic activity, at the same time leading to new questions about the link between eye movements, visual perception and postural control.

## Figures and Tables

**Table 1:** Significant values resulting from the Bonferroni pairwise comparison for the microsaccade peak velocity. Please note that for simplicity only significant comparisons are reported.

Stimulus Pairwise Comparison	*p*-Value
Baseline vs. R_dot	*p* = 0.005
DirR-FixC vs. R_dot	*p* = 0.003
DirR-FixL vs. R_dot	*p* = 0.001
DirL-FixC vs. R_dot	*p* = 0.002
DirL-FixL vs. R_dot	*p* = 0.001
Random vs. R_dot	*p* = 0.03

## Data Availability

Data sharing is not applicable to this article because of the consent provided by participants on the use of confidential data.
